# Maternal Neu5Ac Supplementation During Pregnancy Improves Offspring Learning and Memory Ability in Rats

**DOI:** 10.3389/fnut.2021.641027

**Published:** 2021-10-13

**Authors:** DongSheng Bian, Xinyue Wang, Jiale Huang, Xiaoxuan Chen, Hongwei Li

**Affiliations:** ^1^School of Public Health, Xiamen University, Xiamen, China; ^2^Department of Clinical Nutrition, School of Medicine, Ruijin Hospital Affiliated to Shanghai Jiao Tong University, Shanghai, China; ^3^Department of Clinical Nutrition, Zhongshan Hospital, Fudan University (Xiamen Branch), Xiamen, China

**Keywords:** Neu5Ac, learning and memory, rat pup, HPLC-FLD, offspring

## Abstract

Sialic acids are postulated to improve cognitive abilities. This study aimed to evaluate the effects of sialic acid on behavior when administered in a free form as N-acetylneuraminic acid (Neu5Ac) to pregnant mothers or rat pups. The experiment involved 40 male 21-day-old rat pups and 20 15-day-pregnant rats that were randomized into four Neu5Ac treated groups: 0 (control), or 10, 20, and 40 mg/kg. Morris water maze test and shuttle box test were performed on the rat pups and maternal Neu5Ac-supplemented offspring on day 100 to evaluate their cognitive performance. The Neu5Ac levels in the cerebral cortex and hippocampus were tested with high-performance liquid chromatography-fluorescence detection (HPLC-FLD). We found that the maternal Neu5Ac-supplemented offspring showed better cognitive performance, less escape latency in the Morris water maze test, and less electric shock time shuttle box test, compared with the untreated control. In the meantime, the Neu5Ac level in the cerebral cortex and hippocampus of the offspring was higher in the Neu5Ac treatment group than that in the untreated control group. However, no significant differences were observed between rat pups in the treated and the untreated control groups in terms of cognitive performance and Neu5Ac content in the cerebral cortex and hippocampus. Maternal Neu5Ac supplementation during pregnancy could effectively promote the brain Neu5Ac content of the offspring and enhance their cognitive performance, but Neu5Ac had no such effect on rat pups while directly supplemented with Neu5Ac.

## Introduction

Brain development is a complex process that includes anatomical, biochemical, physiological, and psychological changes. Optimal nutrient supply is essential for the brain to reach its maximum development potential. A balanced intake of protein and energy and the supply of other essential micronutrients are critical for developing the nervous system (1, 2). Nutrition supply in early life affects brain development and determines neurocognitive abilities in adulthood. Failure to provide key nutrients during this critical period of brain development may result in lifelong deficits in brain function despite subsequent nutrient repletion (3).

In the late prenatal and early postnatal periods, babies rely on the dietary intake and milk supply of their mother, or formula milk to meet nutritional needs for optimal growth and development. At this point, besides general nutrients, maternal support also provides highly biologically active ingredients necessary for the continuous development of the brain. Thus, breastfeeding is associated with multiple health benefits. Breastfed infants have lower rates of intestinal and autoimmune diseases and show higher intellectual quotient scores compared with formula-fed infants (4–6). The unique composition of human milk plays a key role in the optimal development of newborns, and sialic acids are considered as one of the components responsible for the multiple benefits (7). They are present in most mammalian milk, mainly conjugated to proteins and oligosaccharides, but also as free monosaccharides. This last group of monosaccharides includes some key core structures, such as N-acetylneuraminic acid (Neu5Ac), which encompass sialic acid molecules (8, 9). Neu5Ac is the only form of sialic acid synthesized by humans and is present in the brain in high amounts (10).

N-acetylneuraminic acid, an essential component for synapse, plays a critical role in neuronal differentiation, growth, and regeneration, supporting synapse transmission, maintaining the normal cell function, and thus affecting learning and memory (11, 12). Early life is a critical window for brain development, not only for rapid proliferation and growth of neural cells but also for increases in synapse connections (8). Thus, adequate early-life nutrition supply, such as Neu5Ac, is crucial to ensure optimal cognitive development in infants and children.

Several studies performed in rodents and pigs showed a relationship between Neu5Ac and cognitive function. In rodent models, an exogenous source of Neu5Ac enhanced the frontal-cortex Neu5Ac content and promoted learning performance (13–15). Piglets fed a Neu5Ac-rich diet from casein glycomacropeptide showed higher cortical glycoprotein Neu5Ac and superior learning and memory outcomes compared with controls fed less sialic acid (12).

The role of Neu5Ac in brain development in pups has received increased attention recently. However, most studies on Neu5Ac to date have focused on the effect of Neu5Ac supplementation on behavioral and cognitive outcomes during the postnatal period. Our study investigated the impact of Neu5Ac supplementation on learning and cognition during pregnancy and lactation and weanling in rats to compare the role of Neu5Ac in brain development.

## Materials and Methods

### Animals and Neu5Ac Supplementation

**Figure 1:**
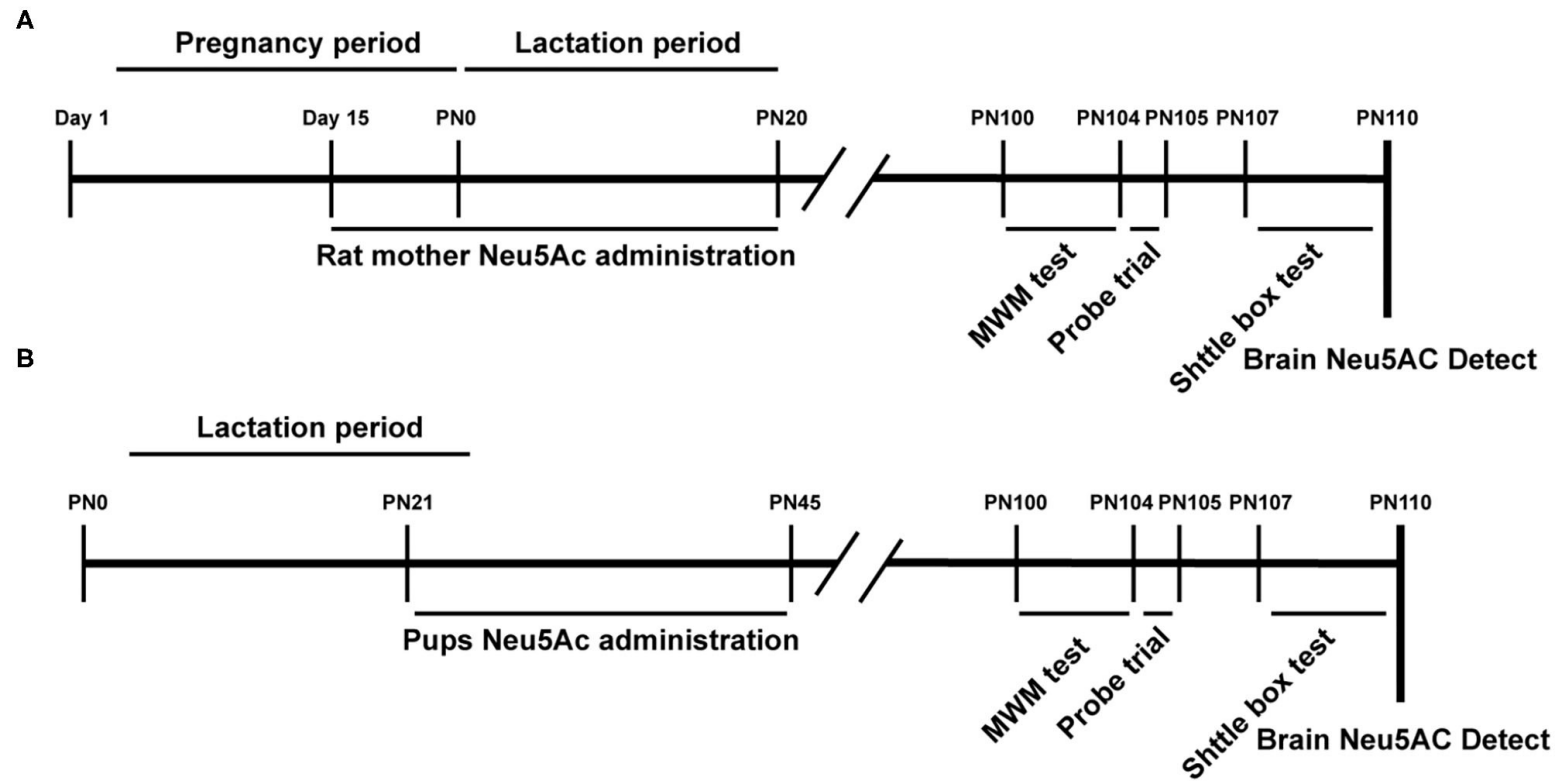
Experimental design scheme. PN, Postnatal day; Neu5Ac, N-acetylneuraminic acid; MWM, Morris Water Maze; shuttle box test. **(A)** Maternal Neu 5Ac supplementation group; **(B)** Rat pups Neu5Ac supplementation group.

A total of 40 male Sprague–Dawley (SD) rat pups (21st day of age) and 40 15-day-old pregnant SD rats (to guarantee that 40 male rat offspring could be obtained) were provided by the Xiamen University Laboratory Animal Center (Xiamen, Fujian, China). The rats were maintained on a 12:12-h light/dark cycle (lights from 07:00 a.m. to 7:00 p.m.) at 22 ± 1°C and relative humidity of 55 ± 10%, with free access to food and water. These rats were randomly divided into four groups, namely, control, low-dose [10 mg/kg · day)], medium-dose [20 mg/kg · day)], and high-dose [40 mg/kg · day)] Neu5Ac-supplemented groups. The rat pup groups were treated with water or Neu5Ac by gavage every day at a set time (11:00 a.m.) for 25 days (Figure 1A). Daily intragastric administration might cause abortion in pregnant rats. Therefore, we selected SD rats at 15 days of gestation; 40 of them were divided into four groups with the indicated doses as earlier (Figure 1B). The mother rats were continuously administered by gavage until the 21st day after birth. On day 21, 10 male offspring from each group were separately raised for further behavioral analysis. On day 100, these rats were subjected to behavioral evaluation.

N-acetylneuraminic acid (assay: 98%) was obtained from Zhejiang ChangXing Pharmaceutical Co. Ltd. (Huzhou, Zheijang, China). All experiments were conducted under the protocols approved by the Animal Care and Use Committee of Xiamen University (code XMULAC20170377).

### Morris Water Maze

Morris Water Maze, developed by Morris, is widely used in behavioral research. Morris Water Maze (MWM) was applied to quantify spatial working or reference memory (16) and visual performance (17). The water maze consisted of a black pool (1.5 m in diameter, 70 cm high, and bottom 50 cm above the floor level) filled with water (made opaque with ink) and a black platform (8 cm in diameter and 45 cm high) submerged below the water surface. The water was maintained at 21 ± 1°C, and the platform was placed in one of the four virtual quadrants 35 cm away from the sidewall. The maze was located in a quiet test room, surrounded by visual cues external to the maze, e.g., an experimenter, colorful signs, a pole, etc., which were visible from within the pool and used by the rats for spatial orientation. Cue locations were not altered during testing. The movement of the rats was recorded using a television camera located over the center of the pool and connected to a personal computer. One day before training, the rats were allowed to swim for 2 min; all these rats underwent four trials each day for four consecutive days, as shown in Figure 1. Each rat was randomly placed at one of the four points during the trials. If the rat failed to find the submerged platform within 90 s, the researcher guided it to the platform to remain for 15 s. A 1-h interval was imposed before the beginning of the next trial for each tested rat. The times that the rats spent in finding the escape latency, swimming speed, and swimming distance were recorded.

The day after the acquisition phase, a so-called “probe trial” was conducted as previously described (18). The rats were allowed to swim freely in the pool for 90 s with the platform removed. The swimming distance around the previous platform, the total distance, and the number of rats crossing the platform position were analyzed. All data were collected automatically *via* a computer using an automated Sony Camera SSC-G213A tracking system (Sony Electronics Inc., NJ, USA), and rat performances were then analyzed using the analytical software Viewer (Biobserve GmbH, Bonn, Germany).

### Shuttle Box Test

A shuttle box 64 × 24 × 34 cm in size was used in this assay. An electric door made of two electric rods was located in the middle of this box. Each rod was 0.9 cm in diameter, and the distance between the two rods was 1.8 cm center to center. A buzzer (80 dB) was used as a conditioned stimulus (CS). The unconditioned stimulus (US) was an electric shock (1.5 mA) applied to the grid floor. The CS lasted a maximum of 10 s, followed immediately by the US lasting a maximum of 10 s. Each trial consisted of a CS for 10 s and the US for 10 s; the US started 10 s after the CS, with a 20-s interval. A learning session of four consecutive days was performed. Each day consisted of 20 trials of acquisition sessions. The rat received a 2-min adaptation period consisting of free ambulation in the shuttle box to be familiarized with the learning environment. At the beginning of the test, the rat was placed in the left compartment of the shuttle box, close to and facing the end wall. If the rat moved to the opposite compartment during the CS, an active avoidance response was recorded automatically. Both CS and US duration depended on the behavior of the rats. If the rat shuttled during the CS presentation, it immediately terminated, and thus the CS was <10 s. If no shuttle occurred during the CS, then the shock was presented until an escape shuttle occurred. Thus, if the rat escaped the US, then the US presentation was <10 s. No minimum CS or US durations were programmed; only maximum durations were programmed. The electric shock time was recorded as the response to the US in the 20 trials each day; a longer response time indicated poor retention, as shown in Figure 1.

### HPLC-FLD of Sialic Acids

After behavioral experiments, all rats were decapitated. Brains were separated from the cranium onto ice using sterile tools to obtain the cortex and hippocampus tissues, which were stored at −80°C until analysis. We selected the left frontal cortex and hippocampus tissues; 100 mg of the hippocampus and 500 mg of the cortex were weighed after homogenization of these tissues. Afterward, the samples were mixed with nine volumes of normal saline homogeneously, to estimate the Neu5Ac content.

#### DMB Reagents

The reagents were as follows: 8 mM dimethyl balenine (DMB), 1.5 M glacial acetic acid, 0.25 M sodium hyposulfite, 0.25 M sodium sulfite, and 0.8 mM 2-mercaptoethanol.

### Sample Preparation

The homogenate (2 ml) was hydrolyzed with 2 ml (0.15 mol/L) of sulfuric acid (H_2_SO_4_) at 80°C for 1.5 h. After cooling down to room temperature, the homogenate was centrifuged at 3,000 *g* for 30 min. All samples were filtered through a 0.22-μm membrane filter (Millipore Corporation, Burlington, MA, USA) and derived with DMB as follows. Briefly, the sample (90 μl) was mixed with DMB (10 μl) reagent and stored for 2.5 h at 50°C in the dark. A 10 μl of the reaction mixture was used for high-performance liquid chromatography (HPLC) analysis.

### HPLC

The major forms of Neu5Ac were analyzed using HPLC-fluorescence detection (FLD) (Agilent 1200 Series; Agilent Technology, Santa Clara, CA, USA). Separation was carried out on a chromatographic column [LiChrosorb RP-18chromatographic column (250 × 4 mm, 5 μm)] obtained from Merck KGaA (Dramstadt, Germany). DMB derivatives of sialic acid were eluted using 7% (*v*/*v*) methanol and 8% (*v*/*v*) acetonitrile in high-quality water for 30 min at a flow rate of 1 ml/min. All injections were given at 30°C. The eluent was monitored for fluorescence at 373 nm (excitation wavelength) and 448 nm (emission wavelength).

### Statistical Analysis

GraphPad Prism 6.01 software package (GraphPad Software, CA, USA) was used for plotting graphs. Behavioral and shuttle box test data from the training period were analyzed using two-way ANOVA followed by Bonferroni's test to determine differences between the groups (16, 19). Differences between control and Neu5Ac treated groups were assessed using one-way ANOVA followed by Bonferroni's *post- hoc* test in sialic acid and probe test data. All data were analyzed in SPSS 13 (SPSS, Inc., Chicago, IL, USA), the significance level was established at *P* < 0.05 for all tests.

## Results

### Maternal Neu5Ac Supplementation Enhanced the Cognitive Performance of the Offspring

**Figure 2:**
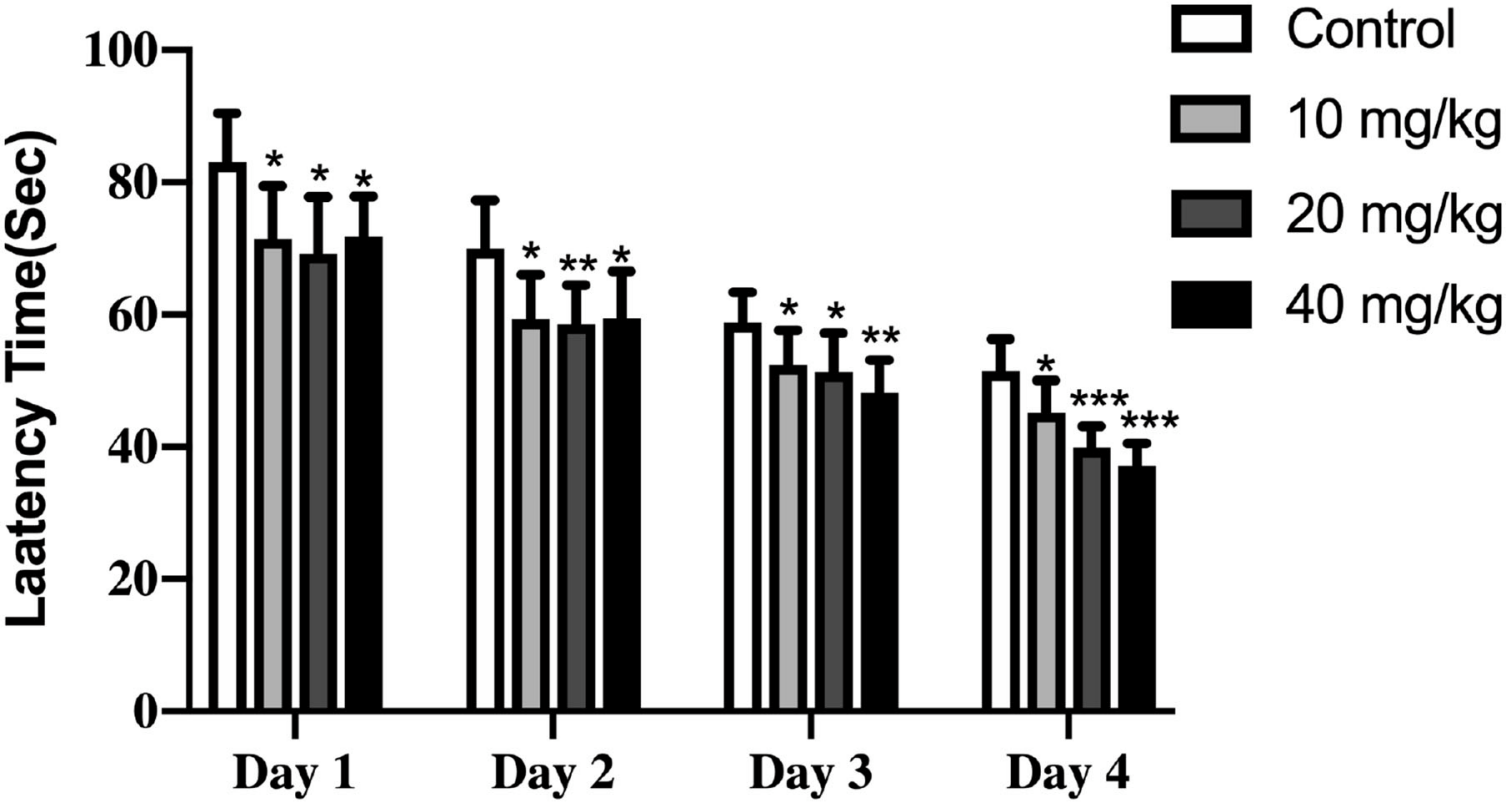
Effects of perinatal Neu5Ac exposure on the latency to find the platform in the Morris water maze test in 100-day-old male rat offspring. Mother rats were treated with Neu5Ac [10, 20, or 40 mg/(kg · day)] or water (as control) from gestational day 15 to postnatal day 21. The results are expressed as the *M* ± *SD*; *n* = 10 per group. *P < 0.05, **P < 0.01, ***P < 0.001 indicate a significant difference compared with control-group rats.

The effect of Neu5Ac on the cognitive performance of rats was determined by supplementing the mothers daily with different doses of Neu5Ac (0, 10, 20, and 40 mg/kg) for 25 days (beginning 15 days after pregnancy till the end of lactation), after which these pups were fed until day 100 to perform the MWM test. First, the rats were trained for four consecutive days to ensure that they were familiar with the pool and the surrounding cues. During the training period, the platform remained in the same position relative to the given clues; these rats must learn to use the clues to localize the platform. Thus, the time that rats spent in finding the platform (latency time) during the training reflected the learning ability in a given pup. As shown in Figure 2, two-way ANOVA revealed that learning differed significantly between the groups and training days [*F*_(9, 108)_ = 2.085; *P* = 0.037]. Moreover, the offspring with their mothers supplemented with Neu5Ac exhibited shorter latency time recorded each day as finding the platform relative to the control groups: day 1 (two-way ANOVA; 10 mg/kg, *P* = 0.013; 20 mg/kg, *P* = 0.01; 40 mg/kg, *P* = 0.01; respectively), day 2 (two-way ANOVA; 10 mg/kg, *P* = 0.01; 20 mg/kg, *P* < 0.01; 40 mg/kg, *P* = 0.013; respectively), day 3 (two-way ANOVA; 10 mg/kg, *P* = 0.03; 20 mg/kg, *P* = 0.018; 40 mg/kg, *P* < 0.001; respectively), and day 4 (two-way ANOVA; 10 mg/kg, *P* = 0.03; 20 mg/kg, *P* < 0.001; 40 mg/kg, *P* < 0.001; respectively). These results suggested that the rats in the Neu5Ac-treated groups could remember where the platform was located in the pool and swim directly to it, which in turn indicated that offspring with Neu5Ac-supplemented mothers had a better learning ability. In the meantime, we found that the learning-promoting effect of Neu5Ac on rat offspring was dose-dependent.

**Figure 3:**
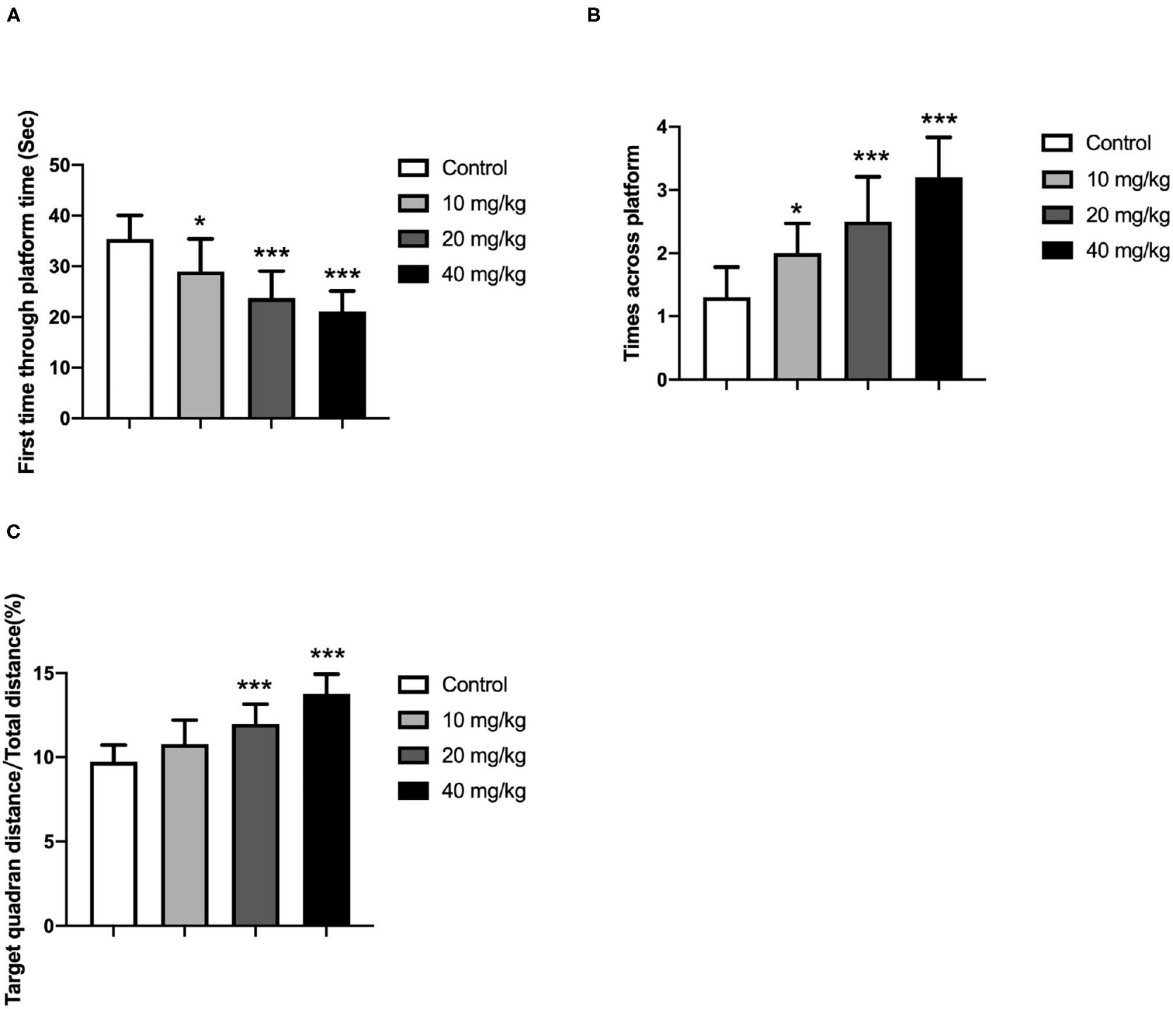
Effects of perinatal Neu5Ac exposure on **(A)** the time spent in the target quadrant; **(B)** the across-platform number; and **(C)** the effective swim distance in the probe test in 100-day-old male rat offspring. Mother rats were treated with Neu5Ac [10, 20, or 40 mg/(kg · day)] or water (as control) from gestational day 15 to postnatal day 21. The results are expressed as the *M* ± *SD*; *n* = 10 per group. *P < 0.05, ***P < 0.001 indicate a significant difference compared with control-group rats.

One day after the training period, these offspring were subjected to a probe trial during which the platform was removed. Additionally, the swimming distance around the previous platform, the total distance, and the number of rats across the platform position were recorded and analyzed because these parameters could be employed to determine the memory ability of these rats. In the probe test, the rats spent more time in the target quadrant on day 5, as indicated in Figures 3A–C (one-way ANOVA). It revealed that Neu5Ac-treated offspring more quickly swam to the platform location [lower first-time-through-platform times; one-way ANOVA, *F*_(3, 36)_ = 21.08; *P* < 0.001; Figure 3A] and were more effective [higher target quadrant distance/total distance; one-way ANOVA, *F*_(3, 36)_ = 14.83; *P* < 0.001; Figure 3C], and moved more times across the platform [one-way ANOVA, *F*_(3, 36)_ = 18.98; *P* < 0.001; Figure 3B] in finding the right location compared with the control groups. This indicated a better memory ability of these Neu5Ac-treated offspring. Taken together, these experiments suggested that maternal Neu5Ac supplementation during pregnancy and lactation could effectively enhance the cognitive performance of the offspring.

**Figure 4:**
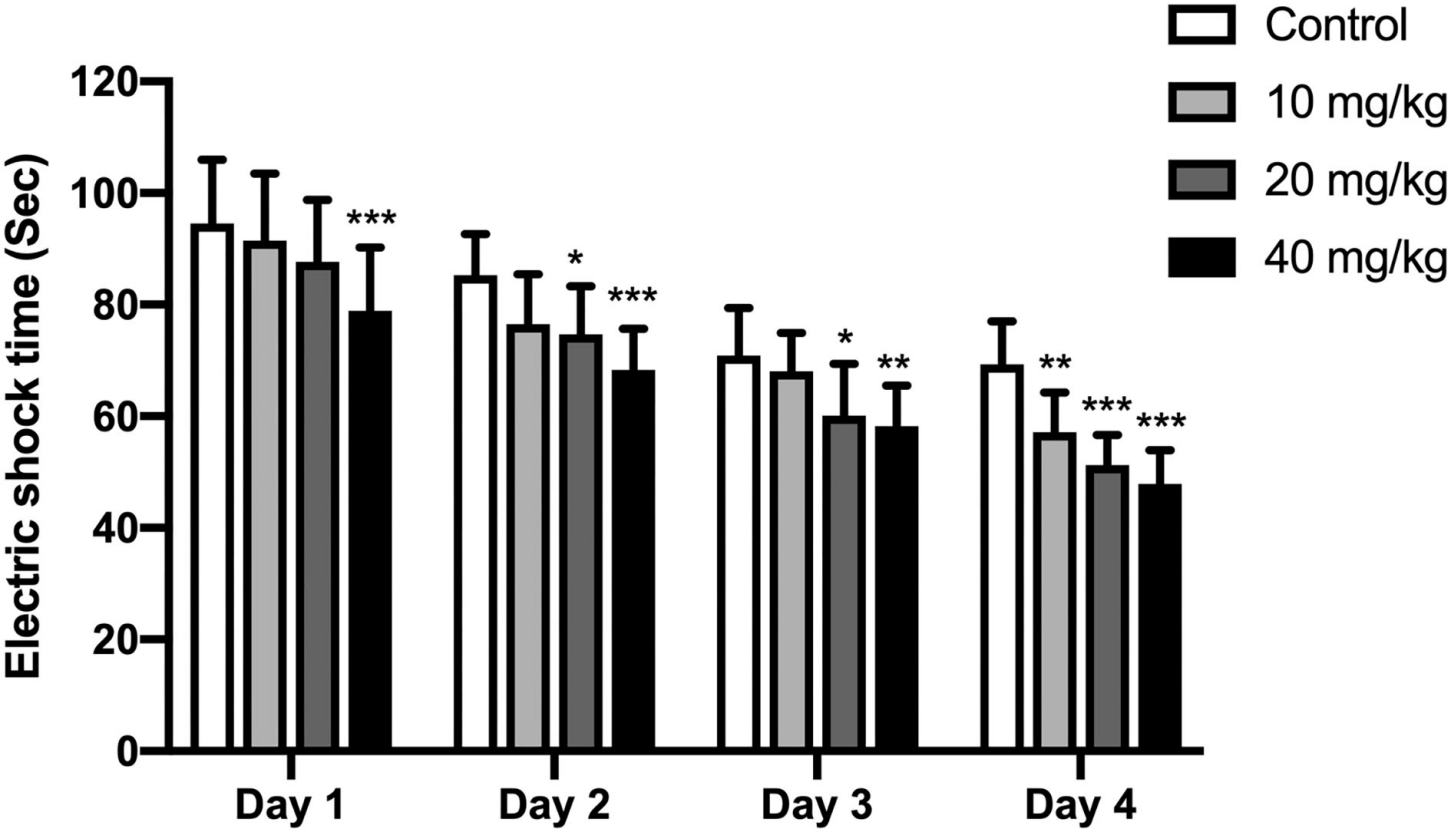
Effects of perinatal Neu5Ac exposure on the electric shock time in the shuttle box test in 100-day-old male rat offspring. Mother rats were treated with Neu5Ac [10, 20, or 40 mg/(kg · day)] or water (as control) from gestational day 15 to postnatal day 21. The shuttle box test was used to evaluate passive avoidance learning in mice following the Morris water maze (MWM) test. The results are expressed as the *M* ± *SD*; *n* = 10 per group. *P < 0.05, ^**^P < 0.01, ***P < 0.001 indicate a significant difference compared with control-group rats.

A shuttle box assay was used to further test the cognitive effect of Neu5Ac. An electric door was located in the middle of this box. At exactly 10 s before the electric shock, a buzzer (80 dB), as the indicator of the electric shock, would be stimulated lasting for 10 s. The offspring were supposed to learn the connection between these cases and avoid the electric shock when the buzzer rang. Thus, the shocking time in these rats could be used to assay their learning and memory ability. As indicated in Figure 4, two-way ANOVA was conducted to investigate the effects of training days and Neu5Ac on electric shock time. The results showed that the time factor [*F*_(3, 108)_ = 96.51; *P* < 0.001] and treatment factor [*F*_(3, 36)_ = 19.14, *P* < 0.001] had an effect on electric shock time in rat offspring. However, no significant interaction was found between treatment and time [*F*_(3, 108)_ = 0.97, *P* = 0.469]. We found that offspring in Neu5Ac-supplemented groups exhibited shorter shock time compared with those in the control group: day 1 (two-way ANOVA, 10 mg/kg, *P* > 0.05; 20 mg/kg, *P* > 0.05; 40 mg/kg, *P* < 0.001; respectively), day 2 (two-way ANOVA; 10 mg/kg, *P* > 0.05; 20 mg/kg, *P* = 0.02; 40 mg/kg, *P* < 0.001; respectively), day 3 (two-way ANOVA; 10 mg/kg, *P* > 0.05; 20 mg/kg, *P* = 0.019; 40 mg/kg, *P* = 0.004; respectively), and day 4 (two-way ANOVA; 10 mg/kg, *P* = 0.006; 20 mg/kg, *P* < 0.001; 40 mg/kg, *P* < 0.001; respectively).

### Maternal Neu5Ac Supplementation Enhanced the Brain Neu5Ac Content of the Offspring

**Figure 5:**
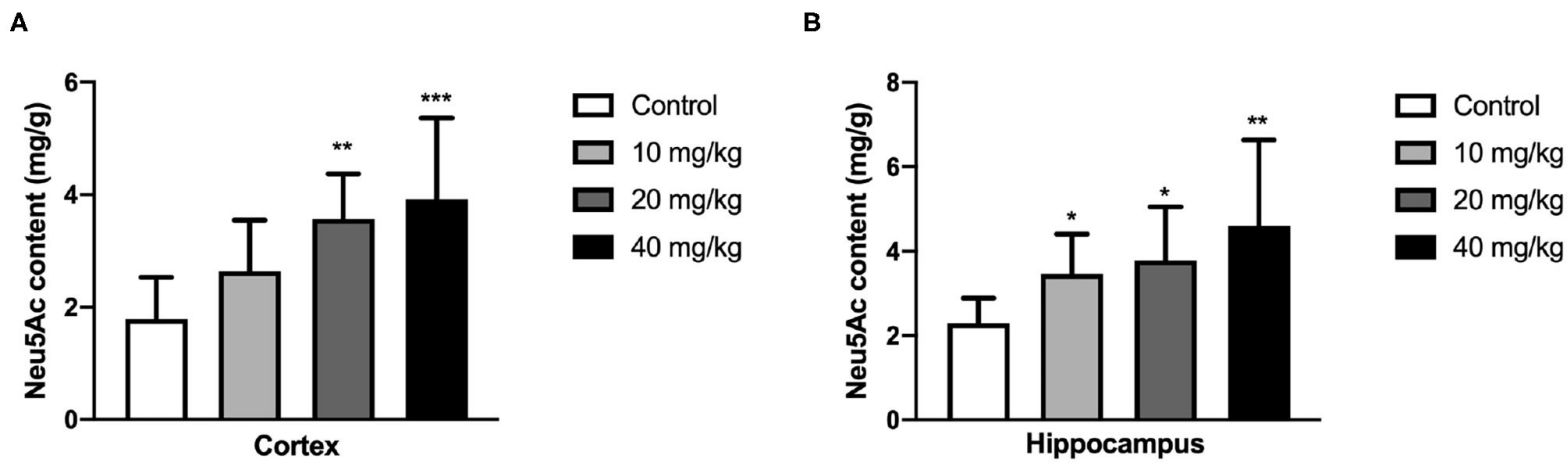
Effects of perinatal Neu5Ac exposure on the brain Neu5Ac content in 100-day-old male rat offspring. Mother rats were treated with Neu5Ac [10, 20, or 40 mg/(kg · day)] or water (as control) from gestational day 15 to postnatal day 21. The results are expressed as the *M* ± *SD*; *n* = 10 per group. *P < 0.05, **P < 0.01, ***P < 0.001 indicates a significant difference compared with control-group rats.

N-acetylneuraminic acid is one of the necessary components in the hippocampus and cortex, and its content is closely related to the development of the hippocampus and cortex and thus the learning and memory. Therefore, the Neu5Ac content in these areas was examined using HPLC assay. As shown in Figure 5, the Neu5Ac content in the hippocampus of the offspring [one-way ANOVA, *F*_(3, 36)_ = 5.279, *P* = 0.004, Figure 5A; 10 mg/kg, *P* = 0.05; 20 mg/kg, *P* = 0.042; 40 mg/kg, *P* = 0.001] and cortex [one-way ANOVA, *F*_(3, 36)_ = 6.790, *P* = 0.001, Figure 5B; 20 mg/kg, *P* = 0.005; 40 mg/kg, *P* < 0.001] with Neu5Ac-supplemented mother was significantly promoted compared with that of the untreated control. This result suggested that maternal Neu5Ac supplementation during pregnancy and lactation could enhance the Neu5Ac content in the hippocampus and cortex of the offspring, which promoted cognitive performance.

### Rat Pups Neu5Ac Supplementation Failed to Promote Adult Cognitive Performance

**Figure 6:**
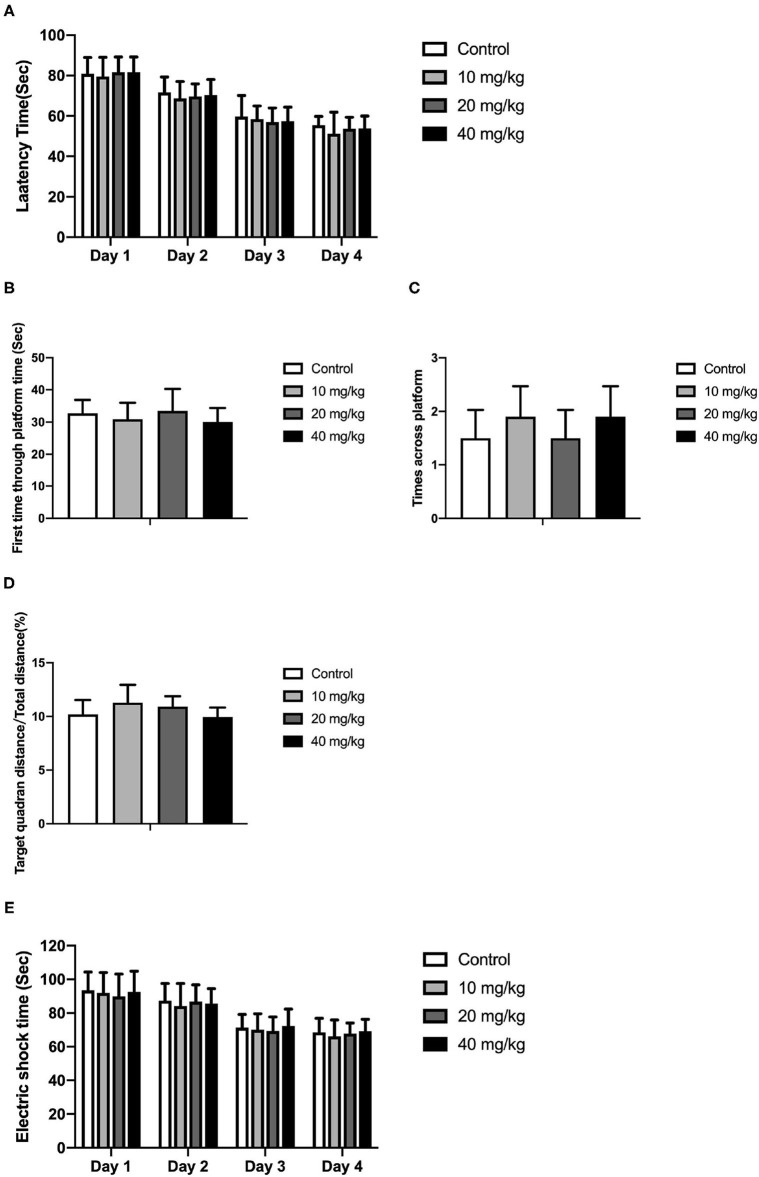
Effects of Neu5Ac exposure on the Morris water maze test and shuttle box test in 100-day-old male rats. These rats were treated with Neu5Ac [10, 20, or 40 mg/(kg · day)] or as control from the 21st day of age to the 45th day of age. **(A)** The latency to find the platform in the Morris water maze test; **(B)** the first-time-through-platform time, **(C)** the across-platform number; and **(D)** the effective swim distance in the probe test. **(E)** Electric shock time in the shuttle box test. The shuttle box test was used to evaluate avoidance learning and memory ability in mice following the Morris water maze (MWM) test. The results are expressed as the *M* ± *SD*; *n* = 10 per group.

**Figure 7:**
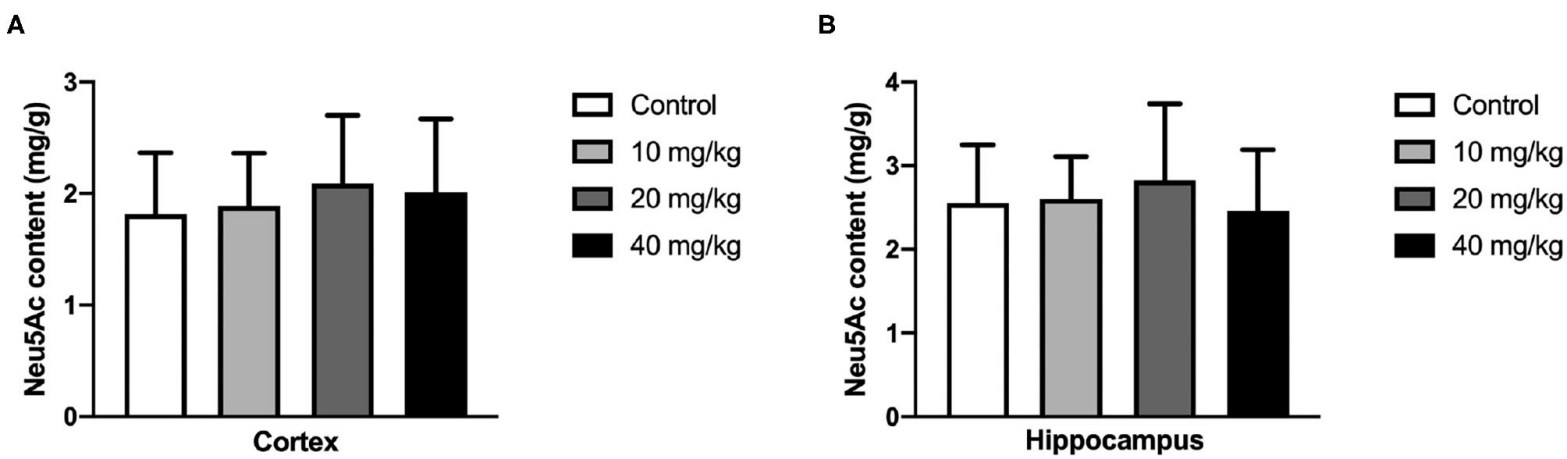
Effects of perinatal Neu5Ac exposure on the brain Neu5Ac content in 100-day-old male rats. These rats were treated with Neu5Ac [10, 20, or 40 mg/(kg · day)] or water (as control) from the 21st day of age to the 45th day of age. The results are expressed as the *M* ± *SD*; *n* = 10 per group.

Most studies focused on the cognitive effect of Neu5Ac supplementation in the early stage of life. However, whether the cognition-promoting effect existed when Neu5Ac was supplemented in later life was still elusive. To clarify this doubt, the newly rat pups (21st day of age) were randomly divided into control, 10, 20, and 40 mg/kg groups, with 10 rats in each group. The rats were administered with different doses (0, 10, 20, and 40 mg/kg) of Neu5Ac for 25 days in the four groups, after which these rats were fed till day 100 and an MWM test and a shuttle box were performed. The two-way ANOVA showed that the time factor [*F*_(3, 108)_ = 96.51; *P* < 0.001] had an effect on the escape latency in rats. No significant interaction was found between treatment and time [*F*_(9, 108)_ = 0.162; *P* > 0.05] (Figure 6A). The multiple comparison test showed that the escape latency had no significant difference between Neu5Ac-treated and control groups (*P* > 0.05). Figures 6B–D shows the first-time-through-platform time [*F*_(3, 36)_ = 0.905; *P* > 0.05], times across the platform [*F*_(3, 36)_ = 1.778; *P* > 0.05], and target quadrant distance/total distance [*F*_(3, 36)_ = 2.476; *P* > 0.05] in the probe test. Moreover, the treatment factor [*F*_(3, 36)_ = 0.646; *P* > 0.05] and the interaction factor [time and treatment, *F*_(9, 108)_ = 0.099; *P* > 0.05] had an effect on the electric shock time (Figure 6E). Neither the MWM test nor the shuttle box assay failed to determine the cognition-promoting effect of Neu5Ac as indicated by the comparable results between the Neu5Ac-treated and the untreated groups. In accordance, the Neu5Ac contents in the rat cortex [one-way ANOVA, *F*_(3, 36)_ = 0.124; *P* > 0.05; Figure 7A] and hippocampus [one-way ANOVA, *F*_(3, 36)_ = 0.448; *P* > 0.05; Figure 7B] showed no significant difference among these groups. These results suggested that later-life, i.e., after the period of rapid brain development, Neu5Ac supplementation had no promoting effect on the cognitive performance of rat pups.

## Discussion

Sialic acids are a family of nine-carbon sugar neuraminic acid, i.e., 5-amino-3,5-dideoxy-D-glycero-D-galactononulsonic acid, which is mostly detected in the mammalian central nervous system (9). It is estimated that 65% of sialic acids are located in gangliosides and 32% in glycoproteins, with the remaining 3% occurring as free sialic acid (20, 21). The neural cell membranes contain 20 times more sialic acid than other types of membranes, suggesting its core role in neural structure. The sialic acid, in the form of glycosphingolipids and one component of gangliosides, is abundant in the cerebral cortex of the human brain (22, 23).

Polysialic acid (polySia) is a linear homopolymer and is most commonly found in the negatively charged α 2-8-linked Neu5Ac residues. The major membrane protein carrier of polySia in mammalian cells is the neural cell adhesion molecule (NCAM). Thus, polySia affects many cellular activities, such as regulating cell migration, axon elongation, neurite outgrowth, and neuronal pathfinding, regeneration, and synaptic formation (24, 25). In addition, PolySia-NCAM is a key neuroplastic molecule, which mediates cell adhesion including neurite fasciculation, neuromuscular interactions, and cell migration thus enabling neuronal plasticity and is instrumental in memory formation (26, 27). As an essential component of polySia, an inadequate supply of Neu5Ac could affect the synthesis of polySia, thus compromising the brain and cognitive development.

The study of Claumarchirant has reported that the total Neu5Ac content in human milk during lactation decreased from the highest level of 136.14 in the early lactation period to the lowest level of 24.47 mg/100 ml in the late lactation period (28). Full-term breastfed infants at 5 months of age possessed nearly twice the amount of total salivary sialic acid compared with formula-fed infants (29) and the brain sialic acid content in breastfed infants was significantly higher than that in formula-fed infants, as observed in babies dying from sudden infant death syndrome (29). The sialic acid levels in brain gangliosides significantly correlated with ganglioside ceramide, docosahexaenoic acid (DHA), eicosapentaenoic acid (EPA), and total (n-3) fatty acids in breastfed, but not in formula-fed, infants. These results suggested that sialic acid, DHA, EPA, and n-3 fatty acids might be functionally linked in the brain of the infant, acting together or synergistically promoting early neurodevelopment and cognition.

The study of Morgan and Winick has demonstrated that the improvement in cognitive ability after administering sialic acid to rat pups correlated with the increased sialic acid levels in the brain, and this benefit was shown to persist into adulthood (30). The study of Wang supplemented a commercial source of sialic acid derived from cheese whey known as casein glycomacropeptide (CGMP), containing 60 mg/g sialic acid, to 3-day-old piglets for 5 weeks. The study found that the piglets fed with higher doses of CGMP showed better performance in learning ability compared with those that fed lower doses of CGMP (12). The study of Karnebeek found that mutations in N-Acetylneuraminate Synthase (NANS), encoding the synthase for Neu5Ac, resulted in a severe developmental delay in infants. Furthermore, this suggested that the requirements for sialic acid in the developing brain must be met at least partially by the endogenous synthesis of sialic acid through the NANS pathway (31). These results indicated that the sialic acid in the brain played a vital role in brain and cognitive development.

In our study, we discovered that Neu5Ac supplementation failed to improve the cognitive ability in rat pups because no significant differences were observed in escape latency among these groups during training. In the meantime, the initial platform crossing time was comparable within these groups in the probe trial. Furthermore, no significant differences were detected in electric shock time between rats in the treated and control groups, and accordingly, the Neu5Ac content in the cerebral cortex and the hippocampus was found to be comparable among these groups. Therefore, we concluded that Neu5Ac supplementation in rat pups could not enhance cognitive ability.

Both the studies of Wang and Morgan showed that Neu5Ac supplementation could promote the cerebral cortex Neu5Ac content and enhance cognitive performance in piglets and rats. However, it should be noted that Neu5Ac administration in these animals was all in the early life, on a postnatal day 3 for piglets and a postnatal day 14 for rats. In rats, the period from fetus to ~21 days of age is characterized by rapid and dynamic brain development and plays an important role in cognitive development. Postnatal day 21 in rats appeared to correspond to a 2- to 3-year-old human child (32). Moreover, the fully matured form of the hippocampus was not developed until weaning (33). Therefore, we speculated that the Neu5Ac supplementation exerted the cognition-promoting function in the early stages of life which is the period of rapid brain development.

Based on the foregoing studies, pregnant SD rats were selected for Neu5Ac supplementation, and the cognitive ability of their offspring was analyzed. We found that the offspring of Neu5Ac-administered rats spent less time escaping the MWM compared with the control ones. Further, they showed less time across the platform compared with the control offspring in the probe trial. Meanwhile, the hippocampus and cerebral cortex Neu5Ac content in the offspring increased along with the increased dosage of Neu5Ac administered to their mother rats. These results suggested that Neu5Ac supplementation during the pregnancy period could enhance the cognitive ability of the offspring. The Neu5Ac content in serum and erythrocyte membranes of pregnant women increased significantly during advanced gestation and persisted for 12 weeks postpartum (34), which was caused by the elevated sialyltransferase activity found in the human placenta (35) to meet the increased sialic acid demand during fetal development. In addition, increased sialic acid synthesis could be detected in the deciduas surrounding the implantation site during early pregnancy, and was associated with the embryo implantation in the uterus of pregnant rats (36). Furthermore, the total Neu5Ac content in the amniotic fluid of healthy pregnant women was found to be significantly increased during pregnancy. Therefore, Neu5Ac that was supplemented to the mother could be delivered to the fetus and, thus enhancing the fetal cognitive development.

A recent study was performed on a knock-out mouse model (B6.129-*St6gal1*^tm2Jxm^/J) lacking the gene responsible for the synthesis of sialyl(alpha2,6)lactose (6′SL), one of the two sources of Neu5Ac to the lactating offspring. It found that lactational 6′SL deprivation affected cognitive capabilities and hippocampal electrophysiology in adulthood. These phenomena were associated with the reduced expression of genes involved in central nervous system development in the hippocampus and prefrontal cortex (37). The biosynthesis of the Neu5Ac occurs mainly in the liver, and GNE(UDP-GlcNAc 2-epimerase) (38) is responsible for Neu5Ac biosynthesis, but it is inadequately expressed in newborn pups and reaches the maximum at postnatal day 15 (39). Wang reported that 3-day-old newborn piglets treated with a diet rich in Neu5Ac for 5 weeks showed a 2- to 3-fold increase in mRNA levels in the hippocampus and liver for GNE and ST8Sia IV (a polysialyltransferase gene, a key gene required for the polysialylation of NCAM) (12). This study suggested that GNE and ST8Sia IV might increase the synthesis of polySia on NCAM in synergy during times of high Neu5Ac demand, learning, and brain growth. PolySia is expressed throughout embryonic brain development and reaches peak expression levels perinatally. During postnatal mouse brain development, the amounts of polySia remain high in the first postnatal week, before they rapidly decline between postnatal days 9 and 17–30% of the level at birth. A steep increase in the levels of ST8Sia II and ST8Sia IV mRNA from E10.5 to E13.5 when plateau levels were reached and maintained until birth. Postnatally, downregulation of the ST8Sia II and ST8Sia IV preceded the decline of polySia (40). These findings demonstrated that sialic acid might be very important in the rapid period of brain development.

Therefore, the infant and the fetus do not have the full endogenous biosynthetic capacity to synthesize the Neu5Ac early enough to meet the growth and developmental needs (28, 31). Our results indicated that exogenously supplemented Neu5Ac could be conveyed by the placenta and milk, which then addressed the demands of the rapidly growing brain. Otherwise, our data suggested that the Neu5Ac supplementation in pregnant rats improved the brain Neu5Ac content in the offspring. More importantly, the spatial learning and memory ability in offspring were also found to be enhanced. However, no promoting effect on spatial learning and memory was observed in Neu5Ac-supplemented rat pups.

## Conclusions

N-acetylneuraminic acid supplementation to the mother rats during pregnancy could enhance the brain Neu5Ac content and promote the cognitive development of the offspring but had no such effect when directly supplemented to the rats who missed the period of rapid brain development.

## Data Availability Statement

The original contributions presented in the study are included in the article/supplementary material, further inquiries can be directed to the corresponding author/s.

## Ethics Statement

The animal study was reviewed and approved by Animal Care and Use Committee (IACUC) of Xiamen University.

## Author Contributions

DB: methodology, formal analysis, experiment, writing of the original draft, and visualization. XW: investigation and data curation. JH: investigation. XC: investigation and writing—review and editing. HL: conceptualization, methodology, visualization, writing—review and editing, and funding acquisition. All authors have read and agreed to the published version of the manuscript.

## Conflict of Interest

The authors declare that the research was conducted in the absence of any commercial or financial relationships that could be construed as a potential conflict of interest.

## Publisher's Note

All claims expressed in this article are solely those of the authors and do not necessarily represent those of their affiliated organizations, or those of the publisher, the editors and the reviewers. Any product that may be evaluated in this article, or claim that may be made by its manufacturer, is not guaranteed or endorsed by the publisher.
